# High source–sink ratio at and after sink capacity formation promotes green stem disorder in soybean

**DOI:** 10.1038/s41598-022-14298-4

**Published:** 2022-06-21

**Authors:** Ryo Yamazaki, Tomoyuki Katsube-Tanaka, Eri Ogiso-Tanaka, Yohei Kawasaki, Tatsuhiko Shiraiwa

**Affiliations:** 1 Western Region Agricultural Research Center (Kinki, Chugoku, and Shikoku Regions), National Agriculture and Food Research Organization (NARO), 6-12-1 Nishifukatsu-cho, Fukuyama-shi, Hiroshima, 721-8514 Japan; 2 Graduate School of Agriculture, Kyoto University, Kitashirakawa Oiwake-cho, Sakyo-ku, Kyoto, 606-8502 Japan; 3 Institute of Crop Science (NICS), National Agriculture and Food Research Organization (NARO), 2-1-2 Kannondai, Tsukuba, Ibaraki 305-8602 Japan

**Keywords:** Light responses, Plant ecology, Agroecology, Biological techniques, Biological models

## Abstract

Green stem disorder (GSD) of soybean is characterized by delayed leaf and stem maturation despite normal pod maturation. Previous studies have suggested that GSD occurrence is promoted by a high source–sink ratio, which is produced by thinning or shade removal at the R5 growth stage (the beginning of seed filling). Here the effects of different times and durations of shade removal after the R5 stage on GSD severity were analyzed. First, shade removal for more than 28 days after R5 increased GSD severity by more than 0.4 point in GSD score. Thinning treatment at R5 increased specific leaf weight by 23%, suppressed stem dry weight reduction, and upregulated 19 genes including those encoding vegetative storage proteins at R5 + 28d, indicating excess source ability relative to sink size. On the contrary, shade removal for 14 days after R5 decreased GSD severity by 0.5 point in GSD score. In this treatment, seed size was smaller, while seed number was significantly larger than control, suggesting that shortage of source ability relative to sink size. These results implied that soybean plants regulate GSD occurrences either positively or negatively according to a source-sink ratio during the R5 to R5 + 28d growth stages.

## Introduction

**Figure 1:**
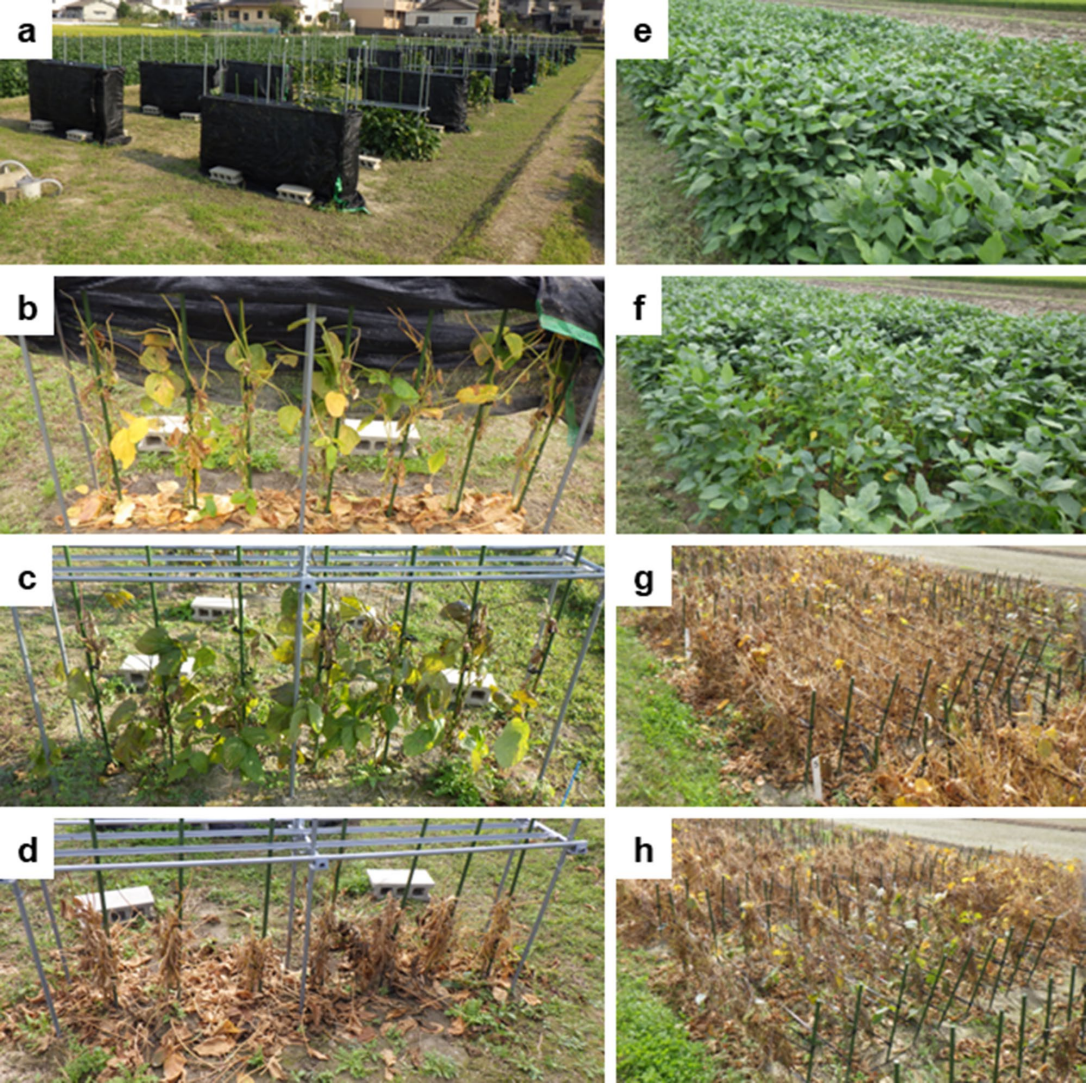
Photographs of the experimental plots in the soybean fields. Soybean cultivation of the shade treatments in Experiments 1 and 2 (**a**–**d**) and the thinning treatments in Experiment 3 (**e**–**h**). (**a**) Photograph on October 3, 2018. (**b**, **c**, **d**) Photographs on the days when the first plant reached the R8 growth stage. (**b**) The full shading (Control), average GSD score of 3.9 on October 26, 2018. (**c**) The shade removal from R5 to R8 (Treatment 3), average GSD score of 4.4 on October 29, 2018. (**d**) The no shade (Treatment 6), average GSD score of 2.2 on October 25, 2018. (**e**, **g**) The dense treatment plot. (**f**, **h**) The thinning treatment plot. (**e**, **f**) R5 growth stage on September 6, 2018. (**g**, **h**) R8 growth stage on November 1, 2018. Photographs were taken by R. Yamazaki.

Green stem disorder (GSD) of soybean (*Glycine max* (L.) Merr.) is a disorder wherein stems and leaves remain green and retain moisture, regardless of mature pods and seeds, at the time of harvesting^1,2^ (medium and sever GSD symptoms were shown in Fig. 1b,c, respectively). GSD is a serious problem for farmers because it negatively affects the harvesting efficiency and seed appearance during combine harvesting of soybean^3–5^. It is important to identify the factors that determine GSD occurrence in soybean^3^.

Previous studies have shown that removing pods (depodding) at the pod setting and filling stages promotes GSD occurrence^6–12^. Depodding resulted in an upregulated gene expression and accumulation of vegetative storage proteins (VSPs) in the stem and leaves^13–15^. Furthermore, other biotic or abiotic factors that possibly promote GSD, such as high soil moisture content and diseases during the reproductive period^16,17^ and pest attack^18^, result in sink limitation at the pod setting and filling stages. According to these studies, a high source–sink ratio induced by sink limitation could result in GSD occurrence^8,13,14^. The same logic was applied in the GSD occurrence promoted by source surplus without sink limitation. Islam et al.^19^ reported that high-N treatments suppressed leaf senescence from the stages R6 (the beginning of full seed stage) to R7 (the beginning of pod maturity) in soybean, resulting in GSD. The number of seeds and pods per plant, representing sink size, was not affected by the N treatments, but seed size was increased. These results are consistent with the hypothesis that the high ratio of source affects GSD.

Furthermore, our previous studies reported that increasing light availability by thinning (after dense cultivation) or shade removal (after side-shading cultivation) at the R5 stage (the beginning of seed filling) increased GSD occurrence in soybean compared to the control plants, which were grown at a dense plant population or were exposed to shading treatments^20,21^. The plants in the thinning treatment at the R5 stage showed more severe GSD symptoms than those in the thinning treatment at the R1 stage (the beginning of flowering)^20,21^. The sink size (seed and pod number) was not significantly affected by thinning or shade removal at the R5 stage, while the sink size significantly increased after these treatments at the R1 stage^20^, suggesting that the R5 stage is critical for determining the source–sink ratio and then GSD.

The period after the R5 stage includes developmentally important events, such as the determination of sink size (seed and pod number)^22,23^, decrease in photosynthetic activity^24^, and the redistribution of assimilates from the stem and leaves to seeds^25,26^. Thus, it is necessary to narrow down the critical or effective growth period after the R5 stage for GSD occurrence under improved light conditions. The objective of this study was to identify the important time and duration of improved light conditions for increased GSD severity and to reveal the effects of light availability on growth parameters and gene expression. First, we employed a side-shade equipment that reversibly altered the assimilation ability of the source by attaching or detaching a shade-sheet to identify the critical growth period for GSD occurrence. Second, plant growth traits and transcriptomes of the stems at the critical growth stage were analyzed under the field conditions, wherein the plant growth habits were different from those in a growth chamber^27^.

## Results

### Effects of shading during the whole growth period on GSD severity and plant growth

Growth traits (seed weight, seed number, stem and pod dry weights) in the no shade showed significantly higher scores (Table 1) than the other five treatments because of different light condition before the R5 growth stage. Since the objective of this study was to see the effects of light improvement after the R5 stage, comparison between the control and treatment 6 was shown just for reference.

**Table 1:** Effects of shading throughout the cultivation period on GSD severity in experiment 1.

Treatment and year		GSD score^a^	Leaf number at R8 (/node)	GSD ratio (%)		Seed weight (g plant^−1^)	Stem dry weight (g plant^−1^)	Pod dry weight (g plant^−1^)	Seed number (plant^−1^)	100 seeds weight (g)	Above ground dry matter (g/plant)/seed number (/plant)^b^
				≤ 3	= 5						
Treatment	Control (shading from sowing to R8)	3.9	0.138	21.4	9.5	27.9	9.9	11.7	82.0	33.8	0.60
	Treatment 6 (shade removal from sowing to R8)	2.6	0.004	85.7	0.0	111.1	30.5	45.9	323.6	34.2	0.58
Year	2017	3.2	0.044	53.6	0.0	68.8	19.2	30.4	209.2	32.8	0.57
	2018	3.3	0.078	53.6	7.1	69.8	20.6	28.0	199.6	34.6	0.60
ANOVA	Treatment	**	**	**	*	**	**	**	**	ns	ns
	Year	ns	ns	ns	ns	ns	ns	ns	ns	ns	ns
	Interaction	ns	ns	ns	ns	ns	ns	ns	ns	ns	ns

**Significantly different (*p* < 0.01). *Significantly different (*p* < 0.05). ns: nonsignificant (*p* < 0.05).

^a^ANOVA was conducted after Box-Cox transformation.

^b^Above ground dry matter (g/plant) means the sum of seed weight (g/plant), pod dry weight (g/plant), and stem dry weight (g/plant).

Statistical analyses were performed using the statistical software BellCurve for Excel version 3.21.

Shading in entire growth periods (full shade) significantly increased GSD severity, with higher GSD score and number of leaves per node at the R8 stage (full pod maturation), and higher percentage of GSD ratio (= 5), and lower percentage of GSD ratio (≤ 3), compared to the treatment 6 (no shade) (Table 1). As an indicator of source-sink ratio, the sum of seed weight, stem and pod dry weights (source ability) per seed number (sink size) was calculated (Table 1). There was no significant difference between the control and treatment 6. There was also no significant difference in seed size (100 seeds weight) or timing of the R8 growth stage (Supplementary Table S1 online) between the two treatments.

### Effects of the time and duration of shade removal on GSD severity

The treatment 1 (shade removal from R5 to R5 + 14 d) showed significantly lower GSD score, fewer number of leaves per node at the R8 stage, and significantly higher GSD ratio (≤ 3) (47.2%) than the control (full shade) (Table 2), suggesting that shade removal from R5 to R5 + 14 d suppressed GSD severity.

**Table 2:** Effects of the time and duration of shade removal on GSD severity.

Treatment and year		GSD score^a^	Leaf number per node at the R8 stage	GSD ratio (%)^b^	
				≤ 3	= 5
Treatment	Control (shading from sowing to R8)	3.9	0.14	21.4	9.5
	Treatment 1 (shade removal from R5 to R5 + 14 d)	3.4*	0.06	47.2*	4.8
	Treatment 2 (shade removal from R5 to R5 + 28 d)	4.3*	0.26**	2.8*	33.3*
	Treatment 3 (shade removal from R5 to R8)	4.5**	0.33**	0.0**	50.8**
	Treatment 4 (shade removal from R5 + 28 d to R8)	3.7	0.09	26.2	2.4
	Treatment 5 (shade removal from R5 + 42 d to R8)	3.7	0.10	33.3	9.5
Year^c^	2017	3.7	0.11	29.4	11.3
	2018	4.0	0.19	18.1	21.4
ANOVA	Treatment	**	**	*	**
	Year	**	**	**	**
	Interaction	ns	ns	ns	*

Dunnett test was used for the comparisons within each column and between the control and other treatments.

**Significantly different (*p* < 0.01). *Significantly different (*p* < 0.05).

^a^ANOVA and Dunnett test were conducted after Box-Cox transformation.

^b^ANOVA and Dunnett test were conducted after angular transformation.

^c^Average score of all treatments in each year.

Statistical analyses were performed using the statistical software BellCurve for Excel version 3.21.

The treatments 2 (shade removal from R5 to R5 + 28 d) and 3 (shade removal from R5 onwards) showed significantly higher GSD score and significantly higher number of leaves per node at the R8 stage than the control (Table 2). The GSD ratio (= 5) was significantly higher in the treatments 2 (33.3%) and 3 (50.8%) than the control (Table 2). However, the GSD severity parameters in the treatments 4 (shade removal from R5 + 28 d onwards) and 5 (shade removal from R5 + 42 d onwards) were not significantly different from the control (Table 2). These results indicated that shade removal for 28 d at and after the R5 stage increased the severity of GSD symptoms, but short-term shade removal treatment, such as 14 d at and after the R5 stage, decreased GSD severity. In contrast, shade removal at and after R5 + 28 d had no effect on GSD severity.

In the timing of R8 growth stage, there were no significant differences between control and each treatment by Tukey’s test, although there were significant differences in treatment and interaction by ANOVA (Supplementary Table S2 online). Thus, the effect of each partial shade treatment on the timing of R8 was not considered in this study.

### Effects of the time and duration of shade removal on growth parameters

Seed number was significantly increased in the shading treatments 1, 2, and 3 compared to the control, treatments 4, and 5 (Table 3). There were no significant differences in the seed number between the treatments 1, 2, and 3, indicating that shade removal from R5 to R5 + 14 d increased seed number, while shade removal after R5 + 14 d had no further effect. Moreover, seed weight significantly increased in the treatments 2 and 3 (Table 3). However, there was no significant difference in the seed weight between the treatments 2 and 3. The treatment 1 showed no significant difference in seed weight, causing a significant decrease in seed size (Table 3). Therefore, seed weight and number were differentially regulated by shade removal during 28 d at and after the R5 stage, while shade removal after R5 + 28 d had no effect on the two growth parameters.

**Table 3:** Effects of the time and duration of shade removal on plant growth parameters.

Treatment and year		Seed weight (g plant^−1^)	Stem dry weight (g plant^−1^)	Pod dry weight (g plant^−1^)	Seed number (plant^−1^)	Seed size (g per 100 seeds)
Treatment	Control (shading from sowing to R8)	27.9b	9.9d	11.7bc	82.0b	33.8a
	Treatment 1 (shade removal from R5 to R5 + 14 d)	33.0ab	10.7cd	14.0b	104.9a	31.5b
	Treatment 2 (shade removal from R5 to R5 + 28 d)	38.7a	12.9bc	16.5a	112.2a	34.4a
	Treatment 3 (shade removal from R5 to R8)	36.2a	15.8a	17.3a	107.9a	33.4ab
	Treatment 4 (shade removal from R5 + 28 d to R8)	26.5b	10.6cd	11.9bc	81.7b	32.4ab
	Treatment 5 (shade removal from R5 + 42 d to R8)	26.4b	9.5d	11.5c	79.1b	33.5ab
Year^a^	2017	31.0	10.5	13.5	97.1	31.8
	2018	31.7	12.1	14.0	93.4	33.8
ANOVA	Treatment	**	**	**	**	*
	Year	ns	**	ns	ns	**
	Interaction	ns	ns	ns	ns	ns

Same letters within a column indicate no significant difference (Tukey's test, *p* < 0.05).

**Significantly different (*p* < 0.01). *Significantly different (*p* < 0.05).

^a^Average score of all treatments in each year.

Statistical analyses were performed using the statistical software BellCurve for Excel version 3.21.

In contrast, stem dry weight was significantly higher in the treatment 3 compared to the control, treatments 1, and 2 (Table 3). Additionally, the stem dry weight of the treatment 2 was significantly higher than the control, suggesting that stem dry weight increased with the duration of shade removal at and after the R5 stage. The treatments 4 and 5 showed no significant differences in stem dry weight.

### Effects of thinning at the R5 growth stage on plant growth

**Figure 2:**
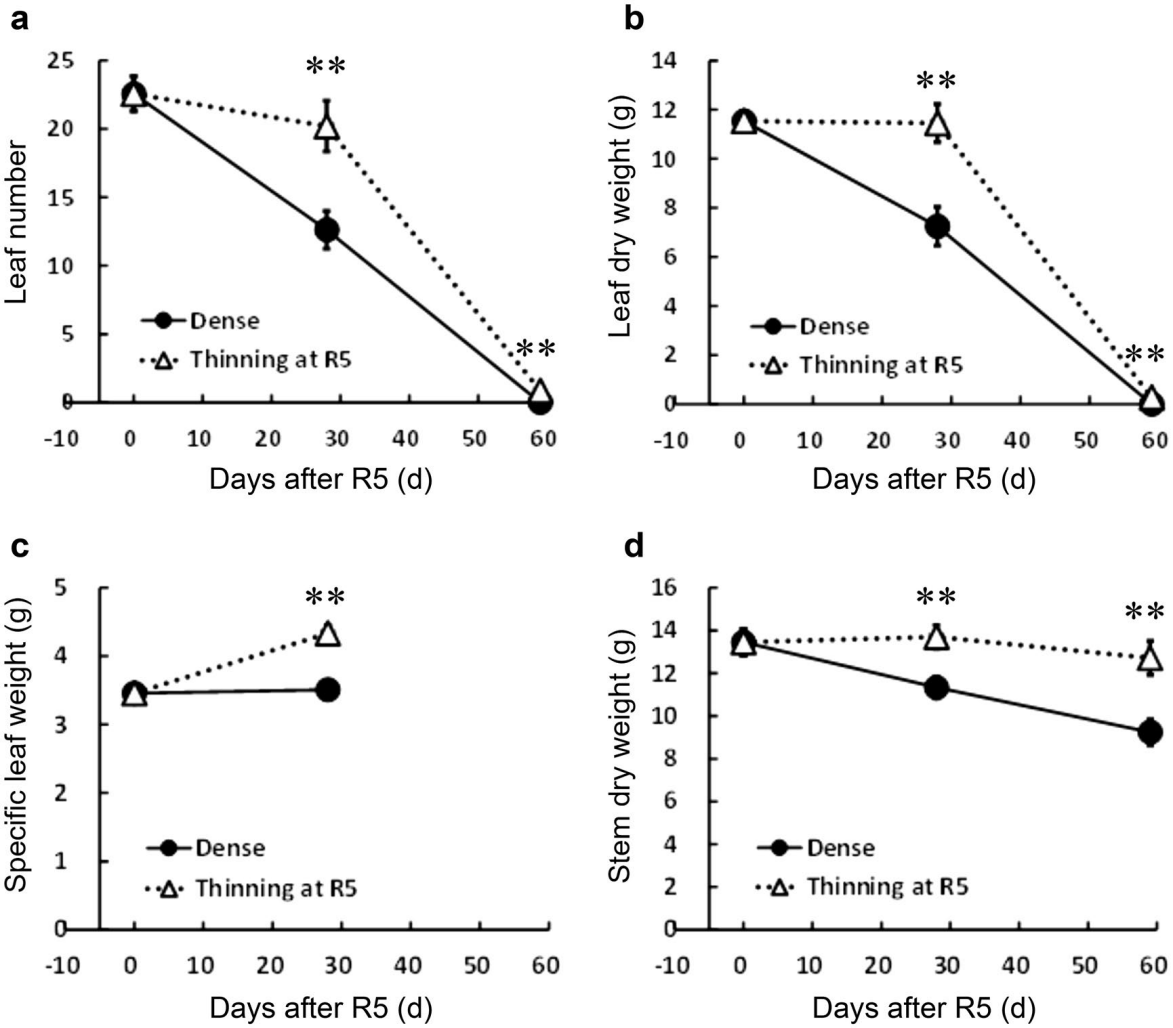
Effects of thinning at the R5 growth stage on growth characteristics. The scale of x-axis indicates days after the R5 stage. (**a**) leaf number, (**b**) leaf dry weight, (**c**) specific leaf weight, and (**d**) stem dry weight. Dense indicates that the plant population was maintained at 22.2 plants m^−2^ throughout the cultivation period. Thinning indicates that the plant population was changed from 22.2 plants m^−2^ to 5.56 plants m^−2^ at the R5 growth stage. The values are the average of two-year experiments. Error bars represent standard error. ** indicates statistically significant difference between dense- and thinning-treatment plots (t-test, *p* < 0.01). Statistical analyses were performed using the statistical software BellCurve for Excel version 3.21.

The thinning treatment at the R5 stage increased GSD severity in the two-year experiments (Table 4, Fig. 1h), which agrees with the results of previous studies^20,21^. After thinning treatment, leaf number decreased from R5 to R8 (Fig. 2a), indicating that leaf abscission progressed after the R5 stage. However, at R5 + 28 d, the number of leaves in the thinning treatment was significantly higher than the dense treatment (Fig. 2a), indicating that the progression of leaf abscission was suppressed by thinning.

**Table 4:** Effects of thinning on GSD severity and plant growth parameters in experiment 3.

Treatment and year		GSD score^a^	Leaf number at R8 per node
Treatment	Dense	1.9	0.001
	Thinning at R5	3.4	0.023
Year	2017	2.6	0.005
	2018	2.7	0.015
ANOVA	Treatment	**	**
	Year	ns	**
	Interaction	ns	**

Statistical analyses were performed using the statistical software BellCurve for Excel version 3.21.

**Significantly different (*p* < 0.01).

*Significantly different (*p* < 0.05).

^a^ANOVA was conducted after Box-Cox transformation.

Although leaf number decreased in both the treatments, the leaf dry weight per plant did not decrease from R5 to R5 + 28 d in the thinning treatment (Fig. 2b) because the leaf dry weight per leaf area (specific leaf weight) increased after thinning, while it remained unaltered in the dense treatment (Fig. 2c). Moreover, the stem dry weight decreased from R5 to R8 in the dense treatment, whereas the decrease in the stem dry weight at R5 + 28 d and R8 was significantly suppressed by thinning (Fig. 2d).

### Effects of thinning at the R5 growth stage on transcriptome

**Figure 3:**
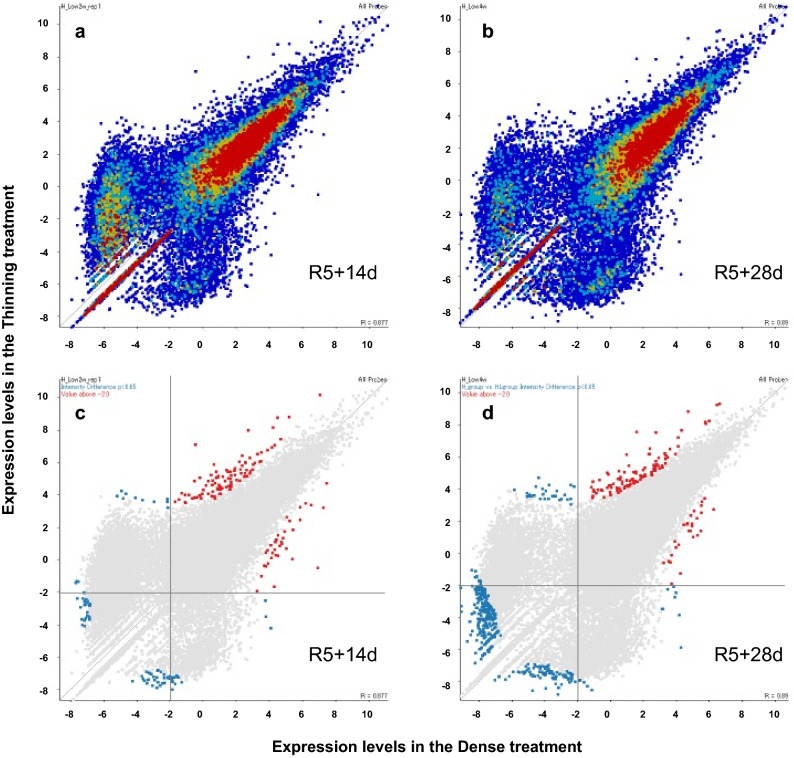
Scatter plots of all the differentially expressed genes (DEGs) in the stem identified by RNA-seq exposed to thinning and dense treatments. Vertical and horizontal axes represent mean expression level in plants exposed to the thinning and the dense treatment, respectively. One plot indicates one gene. (**a**, **b**) Scatter plot of all expressed genes at R5 + 14 d (**a**) and R5 + 28 d (**b**). Warm and cold colors in the plots indicate high and low overlapping levels, respectively. (**c**, **d**) Scatter plots of all DEGs at R5 + 14 d (**c**) and R5 + 28 d (**d**). Colored points indicate DEGs. Red colored points above the horizontal (y =  − 2) and vertical lines (x =  − 2) indicate the genes filtered using intensity difference test (*p* < 0.05) and screened using a logarithmic value of >  − 2.0. Figures were produced with the software SeqMonk v1.38.2 and Java (1.8.0_151).

A total of 54,878 transcriptionally active regions were identified in the stem based on the annotated sequences in the soybean genome. After filtering, the significantly upregulated or downregulated genes were identified in plants exposed to thinning using 211 and 528 probes for the R5 + 14 d and R5 + 28 d datasets, respectively (Fig. 3). The filtered probes were further screened to obtain 126 and 132 probes for the R5 + 14 d and R5 + 28 d datasets, respectively, because the genes with a low expression level scattered quite differently between the dense and thinning treatments (Fig. 3). The analyses of the low expression genes will be reported elsewhere.

GO enrichment analysis showed that thinning-induced differentially expressed genes (DEGs) were mainly associated with photosynthesis (44.8% in the R5 + 14 d and 56.1% in the R5 + 28 d datasets) (Table 5). The second group of genes induced by thinning were involved in carbohydrate metabolism (24.1% in the R5 + 14 d and 9.8% in the R5 + 28 d datasets). However, the GO enrichment analysis of approximately half of the transcriptionally active genes (26,836 genes) with a logarithmic value above -2.0 revealed that the photosynthesis-related genes were not the most overrepresented genes (7.2% in the R5 + 14 d and 6.9% in the R5 + 28 d datasets). This indicated that the thinning treatment markedly altered the expression of photosynthesis-related genes in the stem.

**Table 5:** GO enrichment analysis of significantly upregulated or downregulated genes in plants exposed to the thinning treatment at the R5 growth stage.

Datasets	GO-term	Number of genes	Ratio (%)
R5 + 14 d	Photosynthesis	13	44.8
	Carbohydrate metabolic process	7	24.1
	Biosynthetic process	2	6.9
	Growth	2	6.9
	Lipid metabolic process	1	3.4
	Transport	1	3.4
	Signal transduction	1	3.4
	Multicellular organismal development	1	3.4
	Cellular metabolic process	1	3.4
Total		29	100
R5 + 28 d	Photosynthesis	23	56.1
	Carbohydrate metabolic process	4	9.8
	Cell differentiation	3	7.3
	Growth	3	7.3
	Biosynthetic process	2	4.9
	Multicellular organismal development	2	4.9
	Cell growth	1	2.4
	Cellular metabolic process	1	2.4
	Translation	1	2.4
	Nucleobase, nucleotide and nucleic acid metabolic process	1	2.4
Total		41	100

Gene ontology (GO) enrichment analysis was conducted at the web service of SoyBase (https://www.soybase.org/) with annotations from the Wm82.a2. v1 (Glyma 2.0).

The filtered and screened DEGs were then compared between the R5 + 14 d and R5 + 28 d datasets and a set of common 19 upregulated and 8 downregulated genes were identified in both the datasets (Table 6). Of the 18 upregulated genes, eight genes were related to photosynthesis, including those encoding the 22 kDa-protein of photosystem II (PsbS), chlorophyll a/b-binding protein (CP24), chloroplast pigment-binding protein (CP26), and early light-induced proteins (ELIPs). The remaining four upregulated genes encode VSPs, such as 28 and 31 kDa stem glycoproteins (VspA and VspB) and lipoxygenase. VSPs and lipoxygenase were also upregulated by depodding and accumulated in paravenial mesophyll cells as a temporary storage for N^28^.

**Table 6:** Differentially expressed genes in both R5 + 14 d and R5 + 28 d datasets after the thinning treatment at the R5 growth stage.

Expression	Gmax 2.0 primary protein ID	Top descriptive Uniref100 BLASTP hit
Upregulated	Glyma.04G249700	Photosystem II 22 kDa protein
	Glyma.06G113200	Photosystem II 22 kDa protein
	Glyma.06G143700	Potassium transporter
	Glyma.06G301500	Beta-amylase
	Glyma.07G014500	Stem 28 kDa glycoprotein VSPA
	Glyma.08G074000	Chlorophyll a/b-binding protein CP24
	Glyma.08G138200	Myo-inositol-1-phosphate synthase
	Glyma.08G180000	Chlorophyll a/b-binding protein CP24
	Glyma.08G200100	Stem 31 kDa glycoprotein VSPB
	Glyma.09G154700	Chloroplast pigment-binding protein CP26
	Glyma.10G243800	Early light-induced protein
	Glyma.12G102900	Beta-amylase
	Glyma.12G217300	ADR6 protein
	Glyma.12G217400	Sali3-2
	Glyma.15G026400	Lipoxygenase
	Glyma.15G026500	Lipoxygenase
	Glyma.15G052400	Chlorophyll a/b-binding protein CP24
	Glyma.18G057900	Dihydroflavonol reductase
	Glyma.20G150600	Early light-induced protein
Downregulated	Glyma.03G226000	Endo-1,4-beta-mannanase
	Glyma.06G136600	Two-component response regulator-like PRR73
	Glyma.07G043000	Dormancy/auxin associated family protein
	Glyma.13G322500	Xyloglucan endotraglucosylase/hydrolase
	Glyma.15G186100	Cytidine/deoxycytidylate deaminase
	Glyma.15G228300	NA
	Glyma.16G043200	NAC transcription factor
	Glyma.19G223000	Endo-1,4-beta-mannanase

Upregulated or downregulated indicate that expression levels were significantly upregulated or downregulated in the thinning-treatment plot compared to the dense-treatment plot.

Analysis was performed using the software SeqMonk v1.38.2 and Java (1.8.0_151).

A NAC transcription factor (Glyma.16G043200) gene was downregulated in the R5 + 14 d and R5 + 28 d datasets (Table 6). Amino acid homology analysis using BLASTP revealed that Glyma.16G043200 was a homolog of the NAC domain-containing protein 47 (ANAC047) of *Arabidopsis*, which belongs to the stress-responsive NAC-B (SNAC-B) subfamily^29^. *ANAC047* is a senescence-associated gene that promotes senescence and programmed cell death in leaves^30^. The genes encoding endo-1,4-β-mannanase and xyloglucan endotransglucosylase/hydrolase were also downregulated under thinning treatment (Table 6), which are senescence-associated enzymes in leaf abscission zones and degrade cell wall^31^.

## Discussion

### The methodologies to identify the critical growth period under improved light conditions

Turner et al.^32^ reported that depodding resulted in gene expression patterns derived from both sink-limited effects and stress responses to injuries. Meanwhile, in the present study, thinning treatment probably caused a response to high light stress, irrespective of the increased assimilation ability of the source. Transcriptome analysis revealed that photosynthesis-related genes were significantly altered by the thinning treatment (Table 5); eight out of the 19 upregulated genes were related to photosynthesis, such as those encoding PsbS, CP24, CP26, and ELIPs (Table 6). These proteins absorb excess light energy to prevent photo-oxidative stress caused by reactive oxygen species generated under high light intensities^33–35^. Dihydroflavonol reductase (Table 6), i.e., an anthocyanin synthase, also prevents oxidative stress^36^, suggesting that high light intensity-responsible genes were induced by the thinning treatment.

In contrast, shading treatment throughout the cultivation period (full shade) increased GSD severity as well (Table 1). This phenomenon was also observed in the previous study^20^ although shading strength was different from one another. Shading treatment from sowing to R8 significantly reduced the sink capacity and the assimilation ability of the source by approximately 75% (Table 1). There was no significant difference between the control and treatment 6 in the indicator of source-sink ratio (above ground dry matter/seed number, Table 1). It suggested that GSD promoted by continuous shading had different mechanisms which could not be explained by source-sink ratio, as suggested in the previous study^20^.

An intermediate GSD score of full shade enabled to analyze whether the light availability treatment in experiment 2 promotes or suppresses GSD occurrence. Thus, these characteristics coupled with the reversibility of the shading treatment helped in the identification of the critical period for GSD occurrence between 14 and 28 d after the R5 stage.

### Evidence of high source–sink ratio and delayed senescence at the critical period

Depodding results in a rapid increase in dry weights of leaf^13^ and stem^9,16^, suggesting an alteration in the leaf function from an assimilation organ to a storage organ for excess assimilates. In experiment 3, suppressed leaf abscission, increased leaf specific weight, and reduced loss of stem dry weight (redistribution of assimilates from the stem to seeds) from R5 to R5 + 28 d in the thinning treatment indicated that improved light conditions from R5 to R5 + 28 d resulted in excess assimilation products relative to the sink demand (Fig. 2).

Brown and Hudson^27^ attempted to distinguish the effect of sink limitation from that of injury stress responses by analyzing the transcriptomes of depodded and male-sterile soybean plants. They thought that the common upregulated and downregulated genes in both types of plants were related to the effects of sink-limitation. The results were that lipoxygenase genes were highly expressed in both types of sink-limited plants. Lipoxygenases have distinct roles in stress responses^37^ and as storage proteins^28^. In the present study, four out of the 19 genes upregulated in the stem upon thinning encoded VspA (Glyma.07G014500), VspB (Glyma.08G200100) and two lipoxygenases (Glyma.15G026400 and Glyma.15G026500). These results are consistent with those of Brown and Hudson^27^.

The gene encoding NAC transcription factor (Glyma.16G043200), which was significantly decreased upon thinning in the present study (Table 6), was also suppressed in the leaves of both depodded and male-sterile soybean plants^27^. In soybean, NAC transcription factors GmNAC81 and GmNAC30 promote leaf senescence^38^. As *D* gene in sorghum induces programmed cell death and determines stem water content^39^, Glyma.16G043200 may also promote senescence and programmed cell death in soybean stem and may be related to GSD occurrence.

### GSD severity was affected by source-sink ratio determined by the time and duration of light improvement

**Figure 4:**
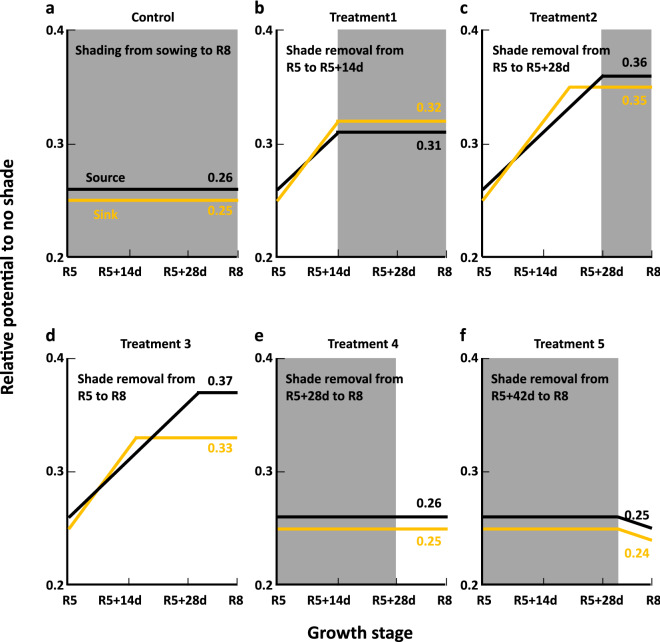
Schematic diagram of relative sink and source potentials to no shade treatment after the R5 growth stage. Control (**a**), treatment 1 (**b**), treatment 2 (**c**), treatment 3 (**d**), treatment 4 (**e**), and treatment 5 (**f**) of Experiment 2. Relative sink potential to no shade was estimated by seed number per plant relative to that of the no shade treatment (treatment 6) at the R8 growth stage (Tables 1, 3). Relative source potential to no shade was estimated by the seed weight, dry weights of pod and stem per plant relative to that of the no shade treatment at the R8 growth stage (Tables 1, 3). In this model, the relative potentials are supposed to be unchanged under shading. The sink potential is increased by shade removal until around R5 + 14d, while the source potential is increased by shade removal until around R5 + 28 d. Shade removal after R5 + 28 d has no or negative effect. The increasing rate of relative potentials under shade removal is supposed to be same between treatments. Gray area indicates the period covered with a shade sheet.

Based on the comparison about the effects of the time and duration of shade removal after the R5 stage on sink size, source ability, and GSD severity, it was indicated that the modulation of the source–sink ratio was associated with GSD severity, which corresponds to the source-sink balance hypothesis. In this context, we proposed a model on the relative potential of source and sink (Fig. 4), which was constructed using the seed number (sink potential) and the seed weight, dry weights of stem and pod (source potential) at the R8 stage in experiments 1 and 2 (Tables 1, 3). The model indicates how the sink and source potentials are determined and how GSD is affected by those potentials. In this model, we employed two assumptions. One is that relative potentials of sink and source were constant during shading. The other one is that the slope (increasing rate) of relative sink and source potentials was identical between treatments during no shade only until a specific grain filling stage, after that the slope becomes zero. The specific stages are around R5 + 14 d and R5 + 28 d for sink and source potential, respectively. For example, the relative potentials of sink and source at R5 were determined by those at R8 in the control and common between the control and treatments 1–5 because the growth before R5 was same between those treatments. Sink size, represented by the seed number, increased in the treatments 1, 2, and 3 compared to the control, while the seed number had no significant difference between the treatments 1, 2, and 3 (Table 3), suggesting that shade removal from R5 to R5 + 14 d determined sink size, while shade removal after R5 + 14 d had almost no further effect on sink size (Fig. 4b,c,d). Similar findings have been reported, in which soybean plants had the ability to adjust sink size in response to light conditions through the abortion of flowers, pods, and seeds until approximately 10–12 or 14–21 d after the R5 stage^22,23^. In contrast, the assimilation ability of the source, represented by the sum of seed weight, dry weights of stem and pod (Table 3), was significantly increased between the treatments 1 and 2 (Fig. 4b,c). Although the treatment 3 showed significantly increased stem dry weight than the treatment 2 (Table 3), there were no significant differences in the seed weight between the treatments 2 and 3. This indicated that an increased supply of assimilates resulting from shade removal from R5 to R5 + 28 d was sufficient to fulfill sink demands, but the sink size did not increase after R5 + 14 d (Fig. 4c,d). Consequently, the balance between the relative potentials of sink and source at R8 determines GSD.

The treatment 1 had significantly smaller seeds and relatively low GSD scores than those of the control treatment (Tables 2, 3). Previous studies reported that source restriction by defoliation or shading reduced seed size^40,41^. Therefore, the reduction in seed size in the treatment 1 suggested shortage of assimilates to fulfill sink demands, which in turn, indicated that shade removal for 14 d after the R5 stage increased sink size without fulfilling sink demands (Fig. 4b).

The photosynthetic ability of soybean decreases with age throughout the pod filling stage^24^. The results of the experiment 3 (Fig. 2) showed that leaf abscission proceeded after the R5 stage and was promoted by mutual shading under a dense plant population, suggesting that the shading treatment in the experiment 2 also promoted the decrease in the photosynthetic activity of plants. Therefore, in the shade removal after R5 + 28 d, plants lost the ability to increase photosynthetic activity in response to improved light conditions (Fig. 4e,f).

It is notable that the increasing rate was higher in the relative potential of sink than that of source under the shade removal until R5 + 14 d (Fig. 4). This model would be further tested to elucidate the molecular mechanism underlying GSD. In the present study, increased GSD severity in soybean upon exposure to improved light conditions at and after the R5 stage was attributed to a high source–sink ratio owing to increased photosynthesis relative to sink size, which was based on the source–sink balance hypothesis.

## Conclusions

Green stem disorder (GSD) of soybean was promoted by a high source–sink ratio, that was increased by either sink limitation or source ability enhancement. Source ability was upregulated by light availability improvement such as thinning and shade removal. The increase in sink and source was, however, possible only by around R5 + 14 d and R5 + 28 d growth stages, respectively. Source and sink abilities could be represented by the sum of seed weight, dry weights of stem and pod and by seed number at maturity, respectively.

## Materials and methods

### Plant material and experimental site

Experiments 1, 2 and 3 were conducted using the soybean cultivar ‘Sachiyutaka’ (Maturity group 6)^42^ in the experimental fields of National Agriculture and Food Research Organization (NARO), Western Region Agricultural Research Center, Hiroshima, Japan (34°30′N, 133°23′E; Elevation: 2 m asl; Soil: Typic fluvaquent). The groundwater level in all the experimental fields was maintained at 30 cm below the surface using the farm-oriented enhancing aquatic system FOEAS^43^, and 3 g m^−2^ N, 10 g m^−2^ P_2_O_5_, and 10 g m^−2^ K_2_O were supplemented to the soil one day before sowing. To check biotic stress, insecticides and fungicides were regularly sprayed in the fields.

### Experiment 1: Shading for the whole growth duration

Seeds were sown on June 28, 2017, and June 29, 2018. The shade equipment in each plot surrounded seven plants in a single row (plant spacing: 0.3 m), without any shade-sheet on the upper part (width: 0.1 m) of the equipment, which mimicked a canopy where some of the uppermost leaves are not shaded by the neighboring plants under field conditions (Fig. 1a)^20^. The shade-sheets used in the equipment were black-colored polypropylene sheets, with a shade strength of 82% (eliminating an average of 82% photosynthetically active radiation in sunlight)^20^ and were raised when one of the uppermost leaves in the plots grew 3 cm taller than the uppermost side of the shade equipment. Thus, the height of the equipment paralleled plant heights throughout the experiment. The shading treatment was applied from sowing to R8 (full shade, control) or never applied (no shade, treatment 6). There were two replicates in 2017 and four replicates in 2018. The main stem of each plant was supported using a gardening pole to prevent lodging (Fig. 1b,c,d).

### Experiment 2: Time and duration of shade removal at and after the R5 growth stage

Five treatments (treatments 1–5) as well as the control treatment were conducted with shading from sowing. The control treatment was common with Experiment 1. In the treatments 1, 2, and 3, shade-sheets were removed at the R5 stage. Then, shading treatment was resumed 14 d and 28 d after the initiation of the R5 stage in the treatments 1 and 2, respectively. The shade-sheets were not resumed after R5 in the treatment 3. In the treatments 4 and 5, shade sheets were removed after 28 d of the R5 stage (R5 + 28 d) and 42 d of the R5 stage (R5 + 42 d), respectively.

### Experiment 3: Thinning at the R5 growth stage

Seeds were sown in 3.0 m × 1.2 m plots on June 27, 2016 (two replicates) and 3.0 m × 2.1 m plots on June 25, 2018 (four replicates). There were two types of plots with different plant population size: dense (Fig. 1e,g) and thinning (Fig. 1f,h) plots. In the dense plots, plant populations were maintained at 22.2 plants m^−2^, with a plant spacing of 0.15 m in 0.3 m rows, throughout the cultivation period. In contrast, the plant populations in the thinning plots were changed from dense to sparse (5.56 plants m^−2^ in 0.6 m rows and 0.3 m plant spacing) at the R5 stage. In the thinning plots, the aboveground parts of the plants in every alternate row and those of every alternate plant in the remaining rows were excised. To prevent lodging, plants were supported by poles standing on both ends of the rows and greenhouse polyethylene bands, which were stretched out between the poles at 40 cm above the ground (Fig. 1g,h).

### Measurements of growth parameters

Seven plants from each plot were sampled at the R8 stage for experiments 1 and 2 during both years. For experiment 3, six (2016) or nine (2018) plants from each dense and thinning treatment plot, except the plants at the plot borders, were randomly selected after the R8 and 28 d of the R5 stage (R5 + 28 d) for the analysis of growth parameters. Moreover, six (2016) or nine (2018) thinned plants, except the plants at the plot borders, were randomly sampled at the R5 stage to analyze the growth parameters of the plants at the start of the thinning treatment.

Growth parameters, including leaf area, leaf number, dry weight of leaflets, and stem dry weight, were measured for each sampled plant of experiment 3. The leaf area was measured using an area meter LI-3100C (LI-COR, Lincoln, NA, USA), and the dry weights of each plant part were measured after drying them at 80 °C for 3 d before measurements.

### Development and GSD score

The dates of the R5 and R8 stages for each plant were determined using the method of Fehr and Caviness^44^. The severity of GSD was determined using the three indexes reported in the previous study, namely GSD score, leaf number per node at the R8 stage, and GSD ratio^20^. GSD score was calculated using a method modified from that of Furuya and Umezaki^45^, and each plant was assigned a GSD score (1–5) based on the number of remaining leaves per node and the color of the stem at the R8 stage, where a high GSD score represented more severe GSD symptoms. In addition, the ratio of the number of plants with no leaves (GSD score: < 3) and the number of plants with one-third of the leaves remaining at the R8 stage (GSD score: 5) to the number of sampled plants were calculated as GSD ratio (≤ 3) and GSD ratio (= 5), respectively for each plot. After the R8 stage, seed weight of 100 seeds and seed number were measured for each sampled plant. Dry weights of the stems and pods were measured after drying them at 80 °C for 3 d.

### Transcriptome analysis

For transcriptome analysis as part of experiment 3, two replicates with three main plant stems, each from dense and thinning treatments after 14 and 28 d of treatment (R5 + 14 d and R5 + 28 d), were collected and freeze-stored in liquid nitrogen at − 80 °C in 2016. Total RNA was extracted using phenol/SDS and treated with DNase I, followed by column purification. The quality and quantity of RNA were ensured using Nanodrop and Agilent 2100 Bioanalyzer, respectively, and RNA-Seq libraries were constructed using Illumina’s Truseq Stranded mRNA LT Sample Prep Kit (as per the manufacturer's instructions) and sequenced using Illumina HiSeq 4000 paired-end sequencing technology, with a read length of 100 and sample size of 7.46 giga base. The sequences were then aligned with the soybean reference genome (Gmax_275_v2.0) using TopHat2 2.0.9^46^.

For the data analysis, filtering and analysis were performed using the software SeqMonk v1.38.2 (http://www.bioinformatics.bbsrc.ac.uk/projects/seand) and Java (1.8.0_151) (https://www.oracle.com/java/). Gene expressions were quantified using the RNA-Seq pipeline quantitation based on merged transcripts, counting reads over exons and correcting for feature length, using log-transformed data. A total of 54,878 transcriptionally active regions (probes) were generated for analysis. However, for the R5 + 14 d dataset, only the first field replicate was used for analysis because of the low RNA quality of the second replicate, whereas both the field replicates of the R5 + 28 d dataset were grouped for analysis. The probes were filtered using the intensity difference test (*p* < 0.05) and then further screened for a logarithmic value (to the base 10) above -2.0. The filtered and screened probes were then analyzed using gene ontology (GO) enrichment analysis of the web service of SoyBase (https://www.soybase.org/) with annotations from the Wm82.a2. v1 (Glyma 2.0).

### Statistical analyses

A completely randomized design was used for each experiment. Statistical significance of differences (*p* < 0.05 or *p* < 0.01) was tested using the analysis of variance (ANOVA) or t-test. Dunnett and Tukey’s tests were used (*p* < 0.05 or *p* < 0.01) to compare the mean values of the control and other treatments and the mean values of plant growth parameters between the treatments, respectively. GSD scores and GSD ratio were analyzed after the Box-Cox and angular transformations, respectively, to meet the assumption that the data be approximately normally distributed. The analyses were performed using the statistical software BellCurve for Excel version 3.21 (Social Survey Research Information Co. Ltd., https://bellcurve.jp/ex/).

### Ethical approval

The soybean cultivar ‘Sachiyutaka’ was provided by National Agriculture and Food Research Organization (NARO). The cultivar, which was developed by NARO and registered as the registration number of 11,367, was used in accordance with the plant variety protection and seed act of Japan. The plant resource can be accessed via NARO Genebank project (https://www.gene.affrc.go.jp/index_en.php). This research was approved by the Western Region Agricultural Research Center, NARO as the research number of 1,040,101, 2016–2018. All the experiments carried out on plants in this study were in compliance with relevant institutional, national, and international guidelines and legislation.

## Supplementary Information

Supplementary Information.

## Data Availability

All data supporting the findings of this study are available within the paper. The transcriptome reads are available in the DDBJ database as accession # DRA008086 under BioProject # PRJDB7775.
