# Supplementary material for: High source–sink ratio at and after sink capacity formation promotes green stem disorder in soybean
Source: Sci Rep. 2022 Jun 21;12:10440. doi: 10.1038/s41598-022-14298-4 (PMC9213405; doi:10.1038/s41598-022-14298-4)
Supplement: Supplementary file 1 — Supplementary Information. [file 41598_2022_14298_MOESM1_ESM.pdf]

Supplementary Tables

High source–sink ratio at and after sink capacity formation promotes green stem disorder in soybean

Ryo Yamazaki, Tomoyuki Katsube-Tanaka*, Eri Ogiso-Tanaka, Yohei Kawasaki, and Tatsuhiko Shiraiwa

Supplementary Table S1. Effects of shading throughout the cultivation period on the R8 growth stage in experiment 1

	Treatment and Year	R8 (days after sowing)
Treatment	Control (shading from sowing to R8)	122
	Treatment 6 (shade removal from sowing to R8)	124
Year	2017	121
	2018	124
ANOVA	Treatment	ns
	Year	ns
	Interaction	ns

ns: nonsignificant ($p < 0.05$).

Statistical analyses were performed using the statistical software BellCurve for Excel version 3.21.

Supplementary Table S2. Effects of the time and duration of shade removal on GSD severity

Treatment and Year		R8 (days after sowing)
Treatment	Control (shading from sowing to R8)	122abc
	Treatment 1 (shade removal from R5 to R5 + 14 d)	121bc
	Treatment 2 (shade removal from R5 to R5 + 28 d)	125a
	Treatment 3 (shade removal from R5 to R8)	123ab
	Treatment 4 (shade removal from R5 + 28 d to R8)	120c
	Treatment 5 (shade removal from R5 + 42 d to R8)	121bc
Year[c]	2017	121
	2018	122
ANOVA	Treatment	*
	Year	ns
	Interaction	**

Same letters within a column indicate no significant difference (Tukey's test, $p < 0.05$).
** Significantly different ($p < 0.01$). *Significantly different ($p < 0.05$). ns: nonsignificant ($p < 0.05$)
Statistical analyses were performed using the statistical software BellCurve for Excel version 3.21.
